# Polycyclic aromatic hydrocarbons (PAHs) contribute to inflammation in a pregnancy cohort

**DOI:** 10.1088/2752-5309/ae6134

**Published:** 2026-05-05

**Authors:** Yoojin Cho, Qi Meng, Kasey E Yu, Irish Del Rosario, Sanjali Mitra, Carla Janzen, Sherin U Devaskar, Beate Ritz

**Affiliations:** 1 Department of Epidemiology, Fielding School of Public Health, University of California, Los Angeles, CA 90095, United States of America; 2 Department of Obstetrics & Gynecology, University of California, Los Angeles, CA 90095, United States of America; 3 Department of Pediatrics, David Geffen School of Medicine, University of California Los Angeles, Los Angeles, CA 90095, United States of America

**Keywords:** polycyclic aromatic hydrocarbons, pregnancy, inflammation, urinary biomarker, cytokine

## Abstract

Polycyclic aromatic hydrocarbons (PAHs) are ubiquitous environmental contaminants generated from incomplete combustion and are detectable in nearly all individuals in the U.S. population. Prenatal PAH exposure has been linked to adverse birth and child health outcomes, but few studies have examined associations between biomarkers of PAHs and inflammation during pregnancy across gestational windows. We investigated associations between urinary PAH metabolites and urinary inflammatory markers among 159 pregnant women enrolled in the placental assessment in response to environmental exposures cohort (2016–2019). Urine samples were collected up to three times during pregnancy (10–17, 18–29, and ⩾30 gestational weeks). Hydroxylated PAHs metabolites were quantified using liquid chromatography–tandem mass spectrometry, and inflammatory markers (IL-6, IL-1*β*, TNF-*α*, and IL-10) were measured using immunoassays. Biomarker concentrations were adjusted for urinary dilution using specific gravity and log-transformation. Effect estimates were generated using linear mixed-effect models with random intercept for each participant to account for repeated measures, and linear regression to assess sampling-period-specific associations while adjusting for maternal age, ethnicity/race, parity, education, and BMI. We found that most PAH metabolites, particularly phenanthrene and naphthalene metabolites, are positively associated with urinary inflammatory markers, except for fluorene metabolites. In mixed-effects models, each doubling of urinary PAHs concentrations was associated with approximately 10%–50% increases in IL-6, IL-1*β*, TNF-*α,* and IL-10 levels. Sampling period-specific analyses indicated that associations with pro-inflammatory cytokines were stronger in early and mid-pregnancy (10–29 weeks), whereas associations with IL-10 were most pronounced later in pregnancy (⩾30 weeks). Results were robust to the exclusion of participants with preeclampsia. These findings indicate that prenatal PAH exposure is associated with sustained inflammatory activity across pregnancy, with gestational timing–specific patterns that may help explain windows of increased vulnerability for adverse pregnancy outcomes. This longitudinal evidence strengthens biologic plausibility linking environmental PAH exposure to maternal inflammatory processes.

## 1. Introduction

Polycyclic aromatic hydrocarbons (PAHs) are generated primarily from incomplete combustion of fossil fuels, biomass, and tobacco [1, 2]. In addition to combustion sources, dietary intake, particularly through consumption of grilled or smoked foods, represents an important exposure pathway in the general population [3, 4]. According to National Health and Nutrition Examination Survey (NHANES) biomonitoring data, urinary PAH metabolites are detectable in the urine of nearly all U.S. individuals (>95%), indicating widespread exposure [5, 6]. Previous studies have linked PAH exposure to reproductive and developmental toxicity in addition to carcinogenicity, respiratory and cardiovascular diseases [7–11]. Here, we will address pregnancy exposures as PAHs can cross the placenta and reach the fetal compartment, potentially affecting both maternal and fetal health [8, 12, 13]. Prenatal PAH exposure has been associated with adverse outcomes such as low birth weight, preterm birth, and impaired neurodevelopment [14–16], with evidence suggesting that early gestational exposure may represent a particularly sensitive window for fetal growth and brain development. Some studies further report increased odds of neural tube defects associated with prenatal exposure, as well as impaired cognitive performance during childhood [17, 18], highlighting the importance of exposure timing during critical stages of embryonic and fetal development. Mechanistically, PAHs undergo metabolic activation that leads to oxidative stress and inflammation, which are key biological pathways implicated in their toxicity [19–21]. These inflammatory and oxidative pathways are central to placental function and immune regulation during pregnancy, and dysregulation of these processes has been implicated in altered fetal development and adverse birth outcomes [22, 23].

Most pregnancy studies focusing on PAHs have examined adverse birth outcomes such as low birth weight, preterm birth, and long-term child health [15, 24]. In contrast, only a limited number of studies have evaluated prenatal PAH exposure in relation to inflammatory or inflammation-related biological markers during pregnancy [25–27]. Existing work has often relied on measurements collected at a single gestational visit or delivery, providing cross-sectional snapshots of exposure–response relationships and limiting the ability to assess how inflammatory patterns evolve across pregnancy. Consequently, longitudinal patterns of inflammatory response to prenatal PAH exposure remain insufficiently characterized.

Here, we examine whether urinary PAH biomarkers, mainly from ambient air pollution sources, are associated with inflammatory responses during pregnancy in a longitudinal study that followed women through pregnancy and collected urine samples repeatedly. This repeated-measures design addresses the limitations of prior single time-point studies by enabling assessment of within-person changes across gestation. Additionally, we aim to characterize temporal patterns of inflammation in relation to biomarkers associated with PAHs exposure to provide further insight into the biological mechanisms that may link PAHs to adverse birth outcomes.

## 2. Methods

### 2.1. Study design and population

This study is based on data collected as part of the Placental Assessment in Response to Environmental Exposures (PARENTs) cohort, a prospective observational study that enrolled 199 pregnant women receiving prenatal care at the University of California, Los Angeles (UCLA) antenatal clinics during 2016–2019. Participants were followed throughout pregnancy, and data was collected at three times (10–17, 18–29, and ⩾30 gestational weeks) under uniform protocols. These sampling windows were defined *a priori* by the original cohort protocol and represent study-specific collection periods rather than clinical trimester. Data was collected through interviews, electronic health records, and biological samples. For 159 PARENTs participants we had at least one recovered and usable urine specimen available for urinary PAH biomarkers and urinary inflammation associated marker analyses at the time the study was approved for participation in the Children’s Health Exposure Analysis Resource (CHEAR) program. We employed complete case analyses restricted to participants with complete covariate data (155 of 159 participants). These participants contributed 391 (out of 450) valid urine samples available. A total of 59 samples were excluded, primarily due to low specific gravity (SG < 1.001; *n* = 48), with additional exclusions for collection during delivery (*n* = 9), missing inflammatory biomarker data (*n* = 1), and unavailable SG information (*n* = 1) (see figure S1 for details). All participants provided written informed consent, and study procedures were approved by the UCLA institutional review board and listed on the ClinicalTrials.gov Website [28].

### 2.2. Biomarker assessment and processing

The pregnancy urine samples we collected were processed immediately upon collection. Samples were centrifuged at 16 000 × g for 10 min at 4 °C to remove residual debris and cells, aliquoted, and stored at −80 °C. They were then shipped on dry ice to the Emory CHEAR laboratory for analysis under standardized protocols. All samples were randomized using a Fisher–Yates shuffling algorithm prior to analysis to reduce any potential batch effects. Randomization was implemented to distribute study samples across analytical runs in a balanced manner while maintaining blinded quality control materials within each batch, and post-analytical review of quality control materials did not indicate systematic batch-related bias. Hydroxylated PAHs metabolites were quantified by liquid chromatography–tandem mass spectrometry and included the sum of 2-hydroxyfluorene and 3-hydroxyfluorene (FLUO2FLUO3), 2-hydroxynaphthalene (NAP2), 1-hydroxyphenanthrene (PHEN1), 2-hydroxyphenanthrene (PHEN2), 3-hydroxyphenanthrene (PHEN3), 4-hydroxyphenanthrene (PHEN4), and 1-hydroxypyrene (PYR1). Urinary inflammation markers were measured using validated immunoassays and included interleukin-6 (IL-6), interleukin-1 beta (IL-1*β*), tumor necrosis factor alpha (TNF-*α*), and interleukin-10 (IL-10). Assay limits of detection were 25 ng l^−1^ for FLUO2FLUO3, 2,500 ng l^−1^ for NAP2, and 12.5 ng l^−1^ for PHEN1, PHEN2, PHEN3, PHEN4, and PYR1, and 1 pg ml for each inflammatory cytokine (IL-6, IL-1*β*, TNF-*α*, and IL-10). Detection frequencies for each biomarker are reported in supplementary table S1. Values below the limit of detection (LOD) were replaced with the LOD/√2 following established practice for handling nondetectable biomarker data [29], and all biomarker concentrations were log-transformed prior to analysis. Quality control using CHEAR pooled materials across batches indicated acceptable assay precision, with coefficients of variation of ∼2%–13% for cytokines and ∼6%–25% for PAH metabolites, with greater uncertainty for results near the detection limit. As both PAHs and inflammation markers were measured in urine, all biomarker concentrations were adjusted for dilution using SG. We used the cohort median SG for the adjustment using the following formula:

\begin{equation*}{\mathrm{Con}}{{\mathrm{c}}_{{\text{SG - adjusted}}}} = {\mathrm{Con}}{{\mathrm{c}}_{{\mathrm{raw}}}} \times \frac{{{\mathrm{S}}{{\mathrm{G}}_{{\mathrm{median}}}} - 1}}{{{\mathrm{S}}{{\mathrm{G}}_i} - 1}}.\end{equation*}

Samples with ${\mathrm{S}}{{\mathrm{G}}_i} &lt; 1.000$ (considered lower than water) were excluded due to measurement error. To reduce the influence of extreme values, we applied 1% winsorization to each biomarker by replacing observations above the 99th percentile with the corresponding percentile thresholds prior to statistical analysis [30].

### 2.3. Statistical analysis

Study population characteristics and distributions of urinary PAHs and urinary inflammation markers were summarized, and patterns across pregnancy examined. First, pairwise correlations among urinary PAHs biomarkers and among urinary inflammation markers were evaluated using Pearson correlation coefficients for exploratory and descriptive purposes (supplementary figures S2 and S3). Then, we employed linear mixed-effects models with a random intercept for each participant that accounted for within-subject correlation arising from repeated urine sampling. Random intercepts were included to account for correlation due to repeated measurements across pregnancy and to capture individual-level baseline heterogeneity in inflammatory marker concentrations. We also assessed associations by sampling period (U1–U3), as defined in the original study design, using multiple linear regression for up to three samples per participants stratified by the sampling period. Sample sizes for each sampling period are reported in supplementary table S2. Covariate adjustment included maternal age (continuous), race/ethnicity (White, non-White), parity (0 or ⩾1), education (⩽high school degree vs 2 year vocational, bachelor’s, master’s, doctorate, professional degree), and body mass index (BMI; categories: underweight, normal, overweight, obese), in the fully adjusted model. The fully adjusted model is presented as the primary analysis to provide effect estimates accounting for all potential confounders (see table S3 and S4 for details). We also conducted a sensitivity analysis excluding samples from participants diagnosed with pre-eclampsia prior to sample collection to assess the robustness of our results. This analysis was done as pre-eclampsia is associated with kidney dysfunction and may change levels of inflammatory biomarkers measured in the urine.

## 3. Results

### 3.1. Study population characteristics

Study population characteristics are shown in table 1. Participants were older (84% ⩾30 years), more highly educated (88% with a bachelor’s degree or higher), and most were non-Hispanic White (46%), followed by Asian (28%), Hispanic (18%), Black or African American (6.9%), and American Indian or Alaskan Native (0.6%). In terms of BMI, 69% of participants were in the normal or underweight range.

**Table 1.** Characteristics of the study population (*N* = 159).

Characteristic	N (%)
Age	

⩽24	3 (1.9%)
25–29	22 (14%)
30–34	77 (48%)
⩾35	57 (36%)

Parity	

0	74 (47%)
⩾1	85 (53%)

Race/ethnicity	

White, non-Hispanic	73 (46%)
Asian	45 (28%)
Hispanic	29 (18%)
Black or African American	11 (6.9%)
American Indian or Alaskan Native	1 (0.6%)

Education	

⩽High school/2-year or vocational	19 (12%)
Bachelor’s	53 (34%)
Master’s	45 (29%)
Doctorate/professional	38 (25%)
Missing	4

BMI	

Underweight	6 (3.8%)
Normal	103 (65%)
Overweight	32 (20%)
Obese	18 (11%)

### 3.2. Urinary biomarker distributions and correlations

Distributions of urinary biomarkers are summarized in table S2. Pearson correlation heatmaps among urinary PAHs and among urinary inflammatory biomarkers across the three sampling periods are presented in figures S2 and S3. Among urinary PAH metabolites (figure S2), correlations were moderate to strong, particularly among phenanthrene metabolites (PHEN1–PHEN4), which showed consistently high correlations in every sampling period. Moderate correlations were also observed between PYR1 and phenanthrene metabolites and between fluorene metabolites and phenanthrene metabolites. Overall, correlation patterns among PAH metabolites were relatively consistent across the three sampling time points. Among inflammatory biomarkers (figure S3), correlations were moderate but consistently positive in every sampling period.

### 3.3. Urinary PAHs biomarkers and inflammatory marker associations

**Figure 1.**
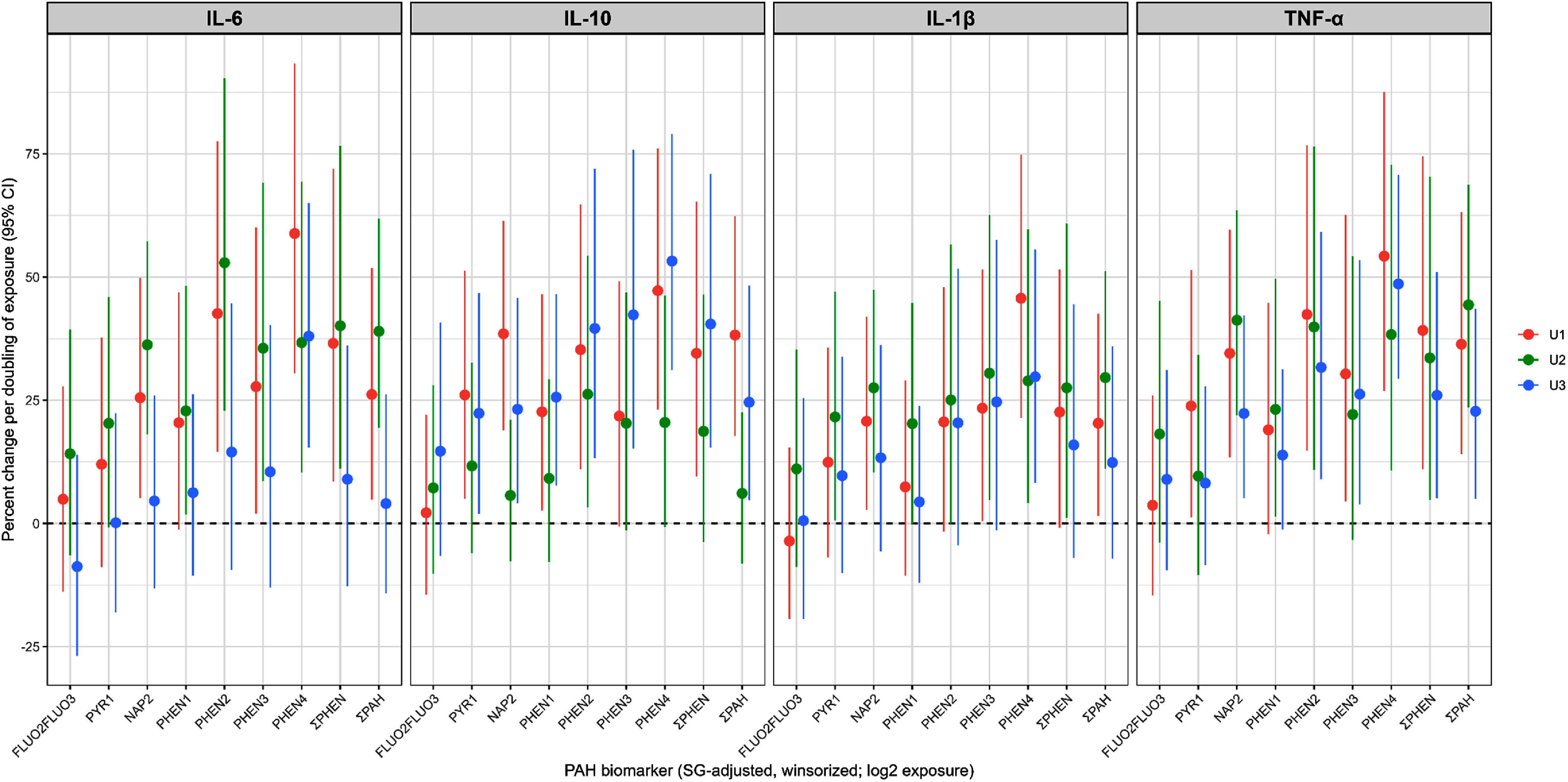
Percent change (and 95% CIs) in inflammatory biomarkers per doubling of urinary PAH exposure (SG-adjusted and log transformed) from fully adjusted linear regression models (adjusted for age, race/ethnicity, parity, education, and BMI across pregnancy sampling periods (U1: 10–17 weeks, U2: 18–29 weeks, U3:⩾30 weeks gestation).

For most PAH metabolites, estimated percent increases in inflammatory markers were positive at all sampling periods. Larger increases in inflammatory markers were observed during the first (U1) and second (U2) urine sampling periods compared to the third sampling period during late pregnancy (U3) (figure 1). This was true for all cytokines except IL-10, for which larger effect sizes were estimated in later pregnancy (U3). Specifically, phenanthrene metabolites and all PAHs combined (ΣPAHs) increased IL-6, IL-1*β* and TNF-*α* levels during early and mid-pregnancy (25%–50% per doubling of PAHs levels) and less strongly in late pregnancy.

**Figure 2.**
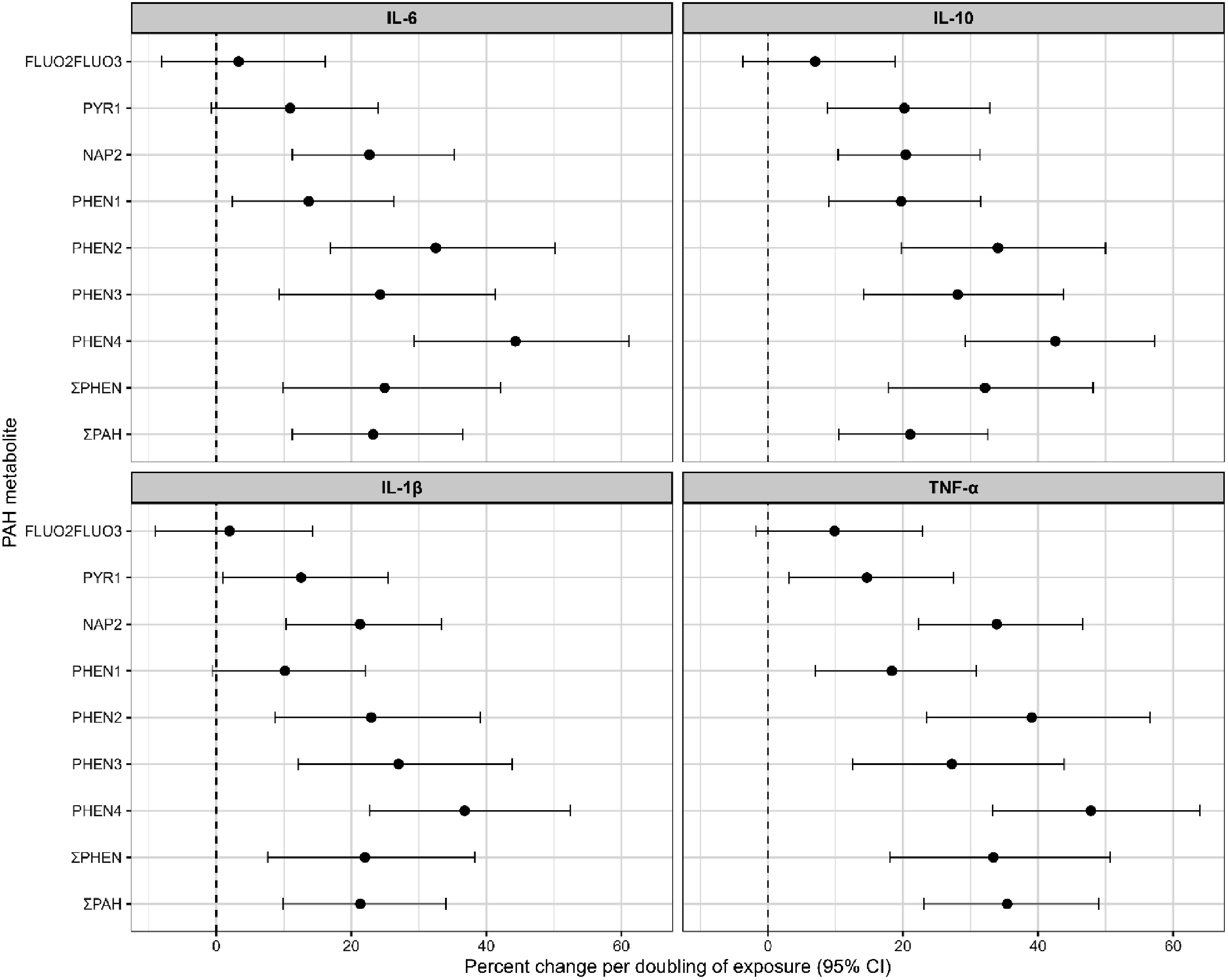
Percent change (and 95% CIs) in inflammatory biomarkers per doubling of urinary PAHs (SG-adjusted and log transformed) from fully adjusted linear mixed effects models (adjusted for age, race/ethnicity, parity, education, and BMI) with random intercepts for participants.

In linear mixed repeated measures models that adjusted for maternal age, ethnicity/race, parity, education, and BMI, each doubling of urinary PAH exposure, except for fluorenes, was associated with higher inflammatory marker concentrations and the 95% CIs excluded the null value i.e. the results were formally statistically significant (figure 2). For IL-10 and TNF-*α*, phenanthrene metabolites (particularly PHEN4), and the PAH summary measure showed the strongest associations (20%–50% increase per doubling of PAH exposure). For example, doubling PHEN4 concentrations was associated with an approximately 45%–50% increase in IL-10 and TNF-*α*, whereas fluorene metabolites (FLUO2FLUO3) showed no formally statistically significant association across inflammatory markers. NAP2 showed a smaller size but statistically significant association. IL-6 and IL-1*β* exhibited similar patterns (10%–40%) with all PAHs except fluorene.

## 4. Discussion

In this prospective cohort of pregnant women, we observed that higher urinary concentrations of PAH metabolites, except fluorenes, were associated with elevated urinary inflammatory markers. The most consistent pattern of increased inflammatory markers was observed for phenanthrene metabolites and the PAH summary measure. These findings align with evidence from other experimental and epidemiological studies that have shown that PAHs can induce systemic inflammation through oxidative stress and activation of pro-inflammatory signaling pathways [20, 31, 32]. Experimental rodent studies provide further evidence for a causal interpretation showing that PAHs exposure leads to inflammatory changes in the placenta [33], which in turn can affect maternal cardiovascular function, uteroplacental blood flow, and the metabolic milieu [34]. Furthermore, these inflammatory changes were also associated with a reduction in rodent fetal weight at mid-gestation, a reduction in late gestation fetal cortical thickness [34], and postnatal changes in the intestinal microbiome [35]. Thus, the inflammatory changes induced by PAHs from air pollution during pregnancy can have far reaching consequences by disrupting the metabolic equipoise in both mother and offspring. PAH metabolites are known to interact with the aryl hydrocarbon receptor, and induce downstream inflammatory signaling pathways, including nuclear factor-*κ*B (NF-*κ*B), and mitogen-activated protein kinase pathways, leading to increased production of cytokines such as IL-1*β*, IL-6, and TNF-*α* [31, 36, 37]. Although IL-10 is generally categorized as an anti-inflammatory cytokine [38], prior studies have shown that IL-10 can also rise as a compensatory regulatory response to oxidative stress-driven inflammation [39, 40]. This feedback role may explain why IL-10 was positively associated with PAH exposure in our cohort throughout and especially late in pregnancy as pregnancy involves dynamic immune modulation, shifting between pro- and anti-inflammatory phases to support implantation, fetal tolerance, and parturition [41]. Taken together, these mechanisms provide biologic plausibility for the positive correlations observed between urinary PAH biomarkers and inflammatory mediators during pregnancy.

Notably, the consistent associations observed among inflammatory biomarkers, phenanthrene, and naphthalene metabolites are supported by prior studies showing that these metabolites exhibit strong metabolic activation potential and induce oxidative stress [7, 8] corroborated by our previous results [42]. However, not all prior human studies reported the same pattern of cytokine associations; for example, some earlier investigations did not observe a link between naphthalene metabolites and TNF-*α*. Such discrepancies may reflect differences in study populations, pregnancy status, exposure distributions, analytical methods, and covariate adjustment strategies, all of which can influence the detection and magnitude of inflammatory responses. In contrast, we did not observe statistically significant associations for fluorene metabolites, even though associations in the positive direction were suggested in the repeated measures models. Several factors may contribute to these null findings. Fluorene metabolites were present at lower concentrations than phenanthrene metabolites in our cohort, which may have reduced statistical power to detect associations. Experimental evidence suggests that fluorene also may exhibit weaker metabolic activation and oxidative stress potential compared to other PAHs, resulting in lower inflammatory signaling activity [43, 44]. These toxicokinetic and toxicodynamic differences may partially explain why fluorene metabolites showed no associations with urinary inflammatory markers in this study. We observed similar patterns in effect estimate magnitude by pregnancy sampling period for all associated PAHs and these patterns likely reflect physiological and immunological adaptations of viable pregnancies resulting in life birth [41, 45, 46].

Inflammatory cytokines are more commonly measured in serum than urine. However, sensitivity analyses excluding participants with preeclampsia did not change our results, suggesting that the associations were not driven by renal dysfunction that are commonly observed in hypertensive pregnancy disorders [47, 48]. Thus, the urinary inflammatory biomarkers we employed likely captured systemic inflammatory responses rather than kidney dysfunction related increases [49–51].

When interpreting these findings, the characteristics of the study population should be considered. Study participants were ethnically and racially diverse, more highly educated, and did not smoke during pregnancy, while also being older and more likely to experience adverse pregnancy outcomes. These factors can all contribute to inflammation and were controlled for in our study. A major strength of our study is the repeated-measure design that allowed assessments throughout gestation. Although the sample size was modest, we had sufficient statistical power to estimate consistent cytokine increases in response to PAH exposures.

## 5. Conclusion

Higher urinary concentrations of PAH metabolites were consistently associated with elevated urinary inflammatory markers during pregnancy, suggesting that systemic inflammation is a biologically plausible pathway linking PAH exposure to adverse pregnancy outcomes. The repeated-measure design strengthens evidence that these associations persist across gestation and may reflect sustained immune perturbation rather than transient responses. Given the widespread presence of PAHs in urban air pollution and the vulnerability of pregnant populations, these findings underscore the importance of reducing environmental exposures during pregnancy. Future studies with larger cohorts and integrated clinical outcomes are needed to clarify how PAH-related inflammatory changes translate into long-term maternal and offspring health risks. Together, our results contribute to a growing body of evidence that environmental PAH exposure represents a modifiable risk factor with implications for reproductive and developmental health.

## Data Availability

The data cannot be made publicly available upon publication because they contain sensitive personal information. The data that support the findings of this study are available upon reasonable request from the authors. Supplementary material 1 available at https://doi.org/10.1088/2752-5309/ae6134/data1.
